# Two Cases of Puerperal Peritonitis

**Published:** 1852-01

**Authors:** J. M. Lawrence

**Affiliations:** Nashville, Tennessee; late resident Pupil at Bellevue Hospital, New York


ART. II.—*Two Cases of Puerperal Peritonitis.* Reported by J. M. LAWRENCE, M. D., of Nashville, Tennessee; late resident Pupil at Bellevue Hospital, New York.

CASE I.—M. W., aged thirty-five years, native of Ireland, cadaverous look, was delivered of her third child, on the 21st of December, at 10 o'clock, A. M. The labor was natural and easy, lasting eight hours.

22d., 8, A. M. Lochial discharge is going on well; pulse 110 per minute; mind uneasy, and bowels not moved since delivery. Ordered castor oil, ℥ iss, which was rejected, and then gave one ounce of salts, which operated well.

2, P. M. She has had a violent chill, which is followed by profuse perspiration, hot skin, and a partial secretion of milk. Her pulse is full, beating 140 per minute; occasional vomiting and delirium. There is tenderness on pressure in the right iliac region. Gave her ten grains of calomel, with five of camphor, and one of opium. The vomiting was relieved by the free use of ice.

6, P. M. The vomiting has returned. The abdomen is slightly tympanitic, tenderness in the right iliac region much increased; great uneasiness of mind; pulse 130, and small. Bled her to approaching syncope, during which the pulse fell to 120, and increased in volume.

At 9, P. M. She is more tranquil, but the pain is intense on pressure. The vomiting is checked.

Repeated same dose of calomel, camphor, and opium, and ordered warm hop fomentation to the abdomen.

3d, 9, A. M. Rested pretty well the latter part of the night, and is more quiet this morning than on yesterday, though the abdomen is more tympanitic, and the tenderness on the surface over the iliac region is greater; pulse 130; patient has had a small discharge from the bowels. Lochia ceased. Ordered one ounce and a half of castor oil, which was rejected from the stomach; and then gave her five grains of calomel, with two of camphor, and half a grain of opium, to be repeated every two hours.

At 2, P. M., her pulse is 120, and her condition is not improved. I applied six leeches to the hypogastrium, and followed them by warm poultices. Continued the calomel and opium.

5, P. M. Bowels were freely moved by an enema of oil and turpentine. Ordered a blister, twelve by twelve, to abdomen, which is to be dressed with mercurial ointment.

24th, 9, A. M. She is much worse; pulse very frequent and feeble; rested badly during the night. She is constantly vomiting a greenish yellow matter.

Died at 3, P. M.

*Autopsy.*—Intestines distended with flatus. The peritoneum is congested and inflamed to a great degree through its entire extent, though more so in the right iliac region. There is also a slight effusion of serum in the peritoneal cavity, but neither lymph nor pus. The uterus is healthy save a slight inflammation of the veins. The other viscera are normal.

Case II.—Josephine A., twenty-two years old, a native of Ireland, and of intemperate habits, was delivered of her first child, December 22d, at 2 o'clock, A. M., during an easy and natural labor, which lasted six hours. There was much anxiety of mind during and after labor.

At 11, A. M., she is doing well, but being apprehensive of peritonitis, I gave her five grains of calomel, with two of camphor, and one of opium, which is to be repeated every two hours. Also gave her a dose of oil.

At 7, P. M., her bowels had been moved freely, and the the lochial discharge was free.

23d, 2, P. M. Had a slight chill, followed by high fever, perspiration and a slight secretion of milk. She was ordered to continue the calomel and opium in half the former doses.

6, P. M. Tenderness in the right iliac region on the surface; pulse 120, and is tense; set her up in the bed, and bled to approaching syncope, taking about twenty ounces of blood. The pulse became fuller immediately, and decreased ten beats.

24th, 9, A. M. She rested pretty well during the night, having been troubled only with slight vomiting, which was checked by ice. The tenderness is increased, and the abdomen is somewhat tympanitic. Her face is expressive of much anxiety, and she is constantly tossing in bed. The lochial discharge is diminished in quanitty. Continued former treatment.

6, P. M. The tenderness and tympanitis are increased; tongue coated; respiration natural; pulse 130, and tense. I bled her again sixteen ounces, which had the effect of rendering the pulse more natural.

25th, 9, A. M. Tolerably quiet through the night; no motion from the bowels, and she complains of still more pain in the abdomen; she vomits occasionally, and the lochia has ceased. Ordered an enema of oil and turpentine, and hotbran fomentations to the belly; also, the eighth of a grain of morphia every hour.

6, P. M. Bowels moved; still vomiting, the matters thrown up now being bilious; skin is cold and wet with perspiration; pulse is very feeble and quick. Ordered brandy to be freely given, and hot bricks to the feet.

26th, 9, A. M. She is much prostrated, and still vomiting the greenish matter; the pulse is barely perceptible, and respiration labored. She died at 3 o'clock, P. M.

*Autopsy.*—On opening the cavity of the abdomen, I found the intestines distended with flatus; effusion of serum, lymph and pus into the abdomen, with recent adhesions. The whole peritoneal coat was injected, presenting an arborescent appearance. The cavity of the uterus had a dark gangrenous look, and fetid odor; no pus in the sinuses; the gall bladder was distended, and there was effusion of serum into the pleural cavity, and into the pericardium.

